# Role of Transient Receptor Potential Vanilloid 4 in Vascular Function

**DOI:** 10.3389/fmolb.2021.677661

**Published:** 2021-04-26

**Authors:** Liangliang Liu, Mengting Guo, Xiaowang Lv, Zhiwei Wang, Jigang Yang, Yanting Li, Fan Yu, Xin Wen, Lei Feng, Tingting Zhou

**Affiliations:** Wuxi School of Medicine, Jiangnan University, Wuxi, China

**Keywords:** TRPV4, vasodilation, vasoconstriction, vascular permeability, vascular remodeling, vascular damage, therapeutic target

## Abstract

Transient receptor potential vanilloid 4 (TRPV4) channels are widely expressed in systemic tissues and can be activated by many stimuli. TRPV4, a Ca^2+^-permeable cation channel, plays an important role in the vasculature and is implicated in the regulation of cardiovascular homeostasis processes such as blood pressure, vascular remodeling, and pulmonary hypertension and edema. Within the vasculature, TRPV4 channels are expressed in smooth muscle cells, endothelial cells, and perivascular nerves. The activation of endothelial TRPV4 contributes to vasodilation involving nitric oxide, prostacyclin, and endothelial-derived hyperpolarizing factor pathways. TRPV4 activation also can directly cause vascular smooth muscle cell hyperpolarization and vasodilation. In addition, TRPV4 activation can evoke constriction in some specific vascular beds or under some pathological conditions. TRPV4 participates in the control of vascular permeability and vascular damage, particularly in the lung capillary endothelial barrier and lung injury. It also participates in vascular remodeling regulation mainly by controlling vasculogenesis and arteriogenesis. This review examines the role of TRPV4 in vascular function, particularly in vascular dilation and constriction, vascular permeability, vascular remodeling, and vascular damage, along with possible mechanisms, and discusses the possibility of targeting TRPV4 for therapy.

## Introduction

Increasing evidence has shown that ion channels play numerous important roles in cell homeostasis, allowing the passage of specific ions (Hubner and Jentsch, 2002; Jentsch et al., 2004). Ion channels are critical for a variety of physiological and pharmacological functions, and their dysfunction can cause channelopathies associated with many kinds of diseases (Liu and Wang, 2019). Transient receptor potential (TRP) channels are non-selective cation channels originally found in *Drosophila melanogaster*, which form a large superfamily of cation channels involved in many sensory and signal transduction processes (Earley and Brayden, 2015). Activated TRPs depolarize the cellular membrane and activate voltage-dependent ion channels, leading to changes in intracellular Ca^2+^ concentrations ([Ca^2+^]_*i*_), playing an important regulatory role in cells (Michel, 2006).

The mammalian TRP channels can be subdivided into six subfamilies according to amino acid sequence homology: vanilloid (TRPV), classical or canonical (TRPC), polycystin (TRPP), melastatin (TRPM), ankyrin (TRPA), and mucolipin (TRPML) (Yin and Kuebler, 2010; Nilius and Owsianik, 2011). The TRP superfamily is implicated in a variety of external or internal stimuli-sensing and transmission functions including taste, smell, vision, pain, temperature, pH, osmotic pressure, mechanical stress, and many endogenous and exogenous ligands (Wong and Yao, 2011). Generally, TRPs reside in the plasma membrane, assembling homo- or hetero-oligomeric polymers into functional channels (Schaefer, 2005; He and Ma, 2016).

Transient receptor potential vanilloids contain six members (TRPV1–TRPV6) involved in many cellular functions (Randhawa and Jaggi, 2015a). TRPV4 is an osmo-mechanosensitive channel, which is permeable to non-selective cations, such as Ca^2+^, Na^+^, and Mg^2+^ ions (Randhawa and Jaggi, 2015b). Structurally, TRPV4 is an 871 amino acid protein with six transmembrane domains, an ion pore between the fifth and sixth transmembrane domains, an NH_2_-terminal ankyrin repeat domain, and a COOH-terminal cytoplasmic domain (Scheraga et al., 2017). The long amino terminus contains a proline-rich domain and a phosphoinositide-binding domain, and the carboxyl terminus contains a TRP box, a Ca^2+^/calmodulin (CaM)-binding region, and a PDZ domain (Goldenberg et al., 2015a). TRPV4 channels are expressed in most tissues, such as the vasculature, lungs, brain, heart, kidneys, salivary glands, liver, bladder, trachea, skin, bone, spleen, testes, dorsal root ganglions, and trigeminal ganglions (Goldenberg et al., 2015a; Randhawa and Jaggi, 2015b; Cui et al., 2020). Cell swelling activates TRPV4 by the phospholipase A2 (PLA2)-dependent generation of arachidonic acid (AA), and its subsequent metabolization to 5,6-epoxyeicosatrienoic acid (5,6-EET) by a cytochrome p450 (CYP) epoxygenase-dependent pathway (Vriens et al., 2004). Phorbol esters and heat activate TRPV4 depending on an aromatic residue at the N terminus of the third transmembrane domain (Vriens et al., 2004), suggesting that stimuli activating TRPV4 promotes channel-opening via distinct pathways. The main endogenous agonists include mechanical flow stimuli, lipid mediator AA (Cao et al., 2018), and its EET metabolites (Kotlikoff, 2005). The synthetic ligands 4α-phorbol 12,13-didecanoate (4α-PDD) (Vriens et al., 2007) and GSK1016790A (Thorneloe et al., 2017) can also activate TRPV4, as well as several natural products such as apigenin (Wei et al., 2017), eugenol (Peixoto-Neves et al., 2015), morin (Zhang et al., 2019), curcumin (Shao et al., 2019), hydroxysafflor yellow A (Yang et al., 2020), omega-3 fatty acid, (Zhu et al., 2020) and puerarin (Zhou et al., 2020). Additional antagonists of TRPV4 include RN1734 (Greenberg et al., 2017), HC067047 (Maqboul and Elsadek, 2018), GSK2193874 (Thorneloe et al., 2017), AB159908 (Arora et al., 2017), and GSK2798745 (Brooks et al., 2019).

**FIGURE 1:**
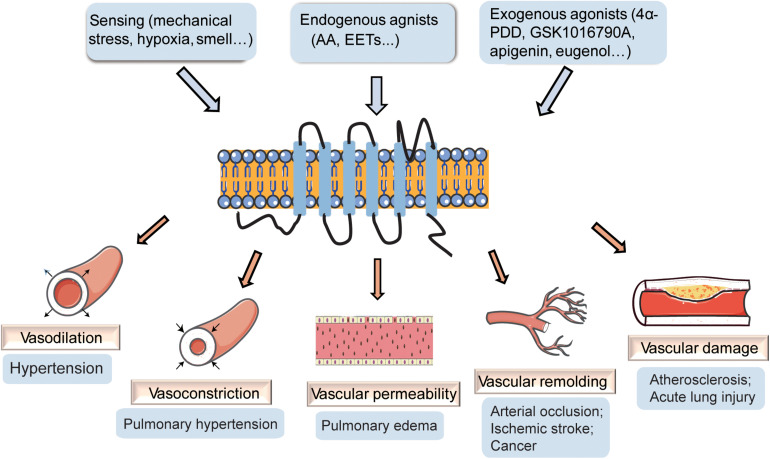
The effect of transient receptor potential vanilloid 4 (TRPV4) on vasculature. TRPV4 channels can be activated by stimuli-sensing, endogenous agonists, and exogenous agonists. The activation of TRPV4 participates in the regulation of vascular dilation and constriction, vascular permeability, vascular remolding, and vascular damage, playing important roles in vascular related diseases.

Transient receptor potential vanilloid 4 (TRPV4) channels have been reported to play vital roles in the regulation of cardiovascular homeostasis and are critically implicated in the regulation of cardiac remodeling, blood pressure, hypoxic preconditioning, pulmonary hypertension, and pulmonary edema (Randhawa and Jaggi, 2015b). TRPV4 channels are present in vascular smooth muscle cells (VSMCs), vascular endothelial cells, and perivascular nerves (Negri et al., 2019), and increasing studies have revealed the role of TRPV4 channels in the maintenance of vascular function. In the current review, we examine the reports on the involvement of TRPV4 in vascular function (Figure 1), particularly in vascular dilation and constriction, vascular permeability, vascular remodeling, and vascular damage, along with possible mechanisms and discuss its potential as a therapeutic target for vascular diseases.

## Transient Receptor Potential Vanilloid 4 and Vasodilation

### Endothelium-Dependent Vasodilation

TRPV4 is expressed in rodent endothelial cells in both large-conductance and small resistance vessels (Filosa et al., 2013). The functional role of endothelial TRPV4 in vascular tone and reactivity has been largely investigated. The activation of TRPV4 contributes to the apparent relaxation of many kinds of vessels, such as mouse mesenteric arteries (Earley et al., 2009), mouse carotid artery (Loot et al., 2008), rat aortic artery (Willette et al., 2008), rat carotid artery, and rat arteria gracilis (Kohler et al., 2006). Recently, a study shows that shear forces enhance endothelial TRPV4 agonist sensitivity and link TRPV4 activation to acetylcholine (ACh)-mediated endothelium-dependent vasodilation in rat cremaster arterioles (Darby et al., 2018). In human coronary arterioles, flow- or AA-induced dilation is mediated by endothelial TRPV4 through Ca^2+^ entry and mitochondrial reactive oxygen species (ROS) signaling (Bubolz et al., 2012; Zheng et al., 2013), demonstrating that endothelial TRPV4 channels are involved in shear stress- and ACh-induced vasodilation (Hartmannsgruber et al., 2007; Zhang et al., 2009). TRPV4-induced vasodilation is almost completely abolished by the inhibition of nitric oxide synthase (NOS) in large vessels and by the inhibition of Ca^2+^-active K^+^ channels in small vessels (Filosa et al., 2013). Endothelial NOS (eNOS) inhibition impedes TRPV4 activator 4α-PDD- and shear stress-induced dilation in rat carotid arteries (Kohler et al., 2006), and the TRPV4 activator GSK1016790A fails to cause vasodilation in mouse aortic rings from eNOS^–/–^ mice (Willette et al., 2008). These studies suggest that eNOS is very important for endothelial TRPV4-regulated vasodilation in large vessels. Both NOS and cyclooxygenase (COX) inhibition reduce 4α-PDD-induced dilation in rat carotid arteries, but NOS and COX inhibition is ineffective in the resistance artery (Filosa et al., 2013). In mouse mesenteric arteries, NOS inhibition and high K^+^ solution block shear stress-induced dilation, whereas COX inhibition has no effect (Baylie and Brayden, 2011), indicating that COX is not required to mesenteric arterial shear stress-induced dilation. Flow-mediated NO and endothelial-derived hyperpolarizing factor (EDHF) production are markedly reduced in the small mesenteric arteries of TRPV4^–/–^ mice (Mendoza et al., 2010). Recently, it is reported that Piezo1 acts upstream of TRPV4 to induce pathological changes in endothelial cells due to shear stress (Swain and Liddle, 2020). ACh-TRPV4 regulates endothelium-dependent vasodilation mainly via EDHF through the induction of intermediate- and small-conductance Ca^2+^-sensitive K^+^ channels (IK_*Ca*_ and SK_*Ca*_) in small mesenteric arteries (Sonkusare et al., 2012), suggesting that Ca^2+^-active K^+^ channels-mediated EDHF is the main pathway involved in TRPV4-regulated vasodilation in small vessels. Therefore, endothelial TRPV4-mediated vasodilation mainly via NO and EDHF production, and the relative contribution of each varies in different vessel types.

Structurally, TRPV4 channels can interact with several proteins including other members of the TRP channel family or Ca^2+^-sensitive K^+^ channels to form functional complexes (Schaefer, 2005; He and Ma, 2016). The stimulation of Ca^2+^-sensing receptors activates heteromeric TRPV4-TRPC1 channels to induce endothelium-dependent vasorelaxation in rabbit and mouse mesenteric arteries (Greenberg et al., 2019). It is well known that most Ca^2+^-handling proteins from endothelial and SMCs are located in caveolae (Absi et al., 2007; Riddle et al., 2011), where Ca^2+^-relevant signal transduction starts (Isshiki and Anderson, 2003). Caveolar integrity is reported to be essential for PLA2-dependent EDHF signaling in porcine arteries (Graziani et al., 2004). The interaction between caveolin-1 (a structural caveolar protein) and TRPV4 is functionally important for 4α-PDD-induced Ca^2+^ increase and EDHF-mediated relaxation (Saliez et al., 2008). TRPV4-K_*Ca*_2.3 complexes in caveolae play important roles in mouse mesenteric dilation (Goedicke-Fritz et al., 2015; He et al., 2017). These studies suggest the importance of caveolar microdomains in TRPV4-mediated vasodilation.

Additionally, endothelial TRPV4 channels are important for cerebral arteriole dilation (Zhang et al., 2013). The inhibition of TRPV4 and PLA2 partially reduce uridine triphosphate-induced Ca^2+^ influx in the endothelial cells of pressurized rat middle cerebral arteries (Marrelli et al., 2007). TRPV4−mediated Ca^2+^ influx in astrocytic endfeet and mouse neurons results in the recruitment of inositol 1,4,5-trisphosphate receptors (IP3Rs) and Ca^2+^−induced Ca^2+^ release (Dunn et al., 2013). Given that SK_*Ca*_ and IK_*Ca*_ channels are involved in modulating the basal tone in mouse parenchymal arterioles (Cipolla et al., 2009), TRPV4 channels probably influence ACh- and EDH-mediated dilation through IK_*Ca*_ and SK_*Ca*_ channel activation in the endothelial cells of cerebral arterioles (Diaz-Otero et al., 2018).

Taken together, the activation of endothelial TRPV4 induces the production of NO, prostaglandin (PGI_2_) and EDHF to regulate vascular dilation (Baylie and Brayden, 2011), and the effect seems to be dependent upon the vascular bed type. The reason probably due to that spatial organization of signaling elements in differential arteries determines selectively underlying mechanism for vasodilatation regulation (Ottolini et al., 2020).

### Endothelium-Independent Vasodilation

TRPV4 channels are also widely expressed in the smooth muscle cells of various artery types from many species (Filosa et al., 2013). The functional coupling of smooth muscle TRPV4 with large-conductance Ca^2+^-activated K^+^ (K_*Ca*_1.1) is implicated in vascular tone control. For example, in rat cerebral arteries, EETs activate TRPV4 in SMCs and induce Ca^2+^ entry-stimulated Ca^2+^ release from ryanodine receptors (RyR) in the sarcoplasmic reticulum (SR), contributing to Ca^2+^ sparks and subsequently activating K_*Ca*_1.1 to stimulate smooth muscle hyperpolarization and vasodilation (Earley et al., 2005). Fawn hooded hypertensive rat cerebral arterial myocytes display a stretch-sensitive TRPV4-like single-channel current and its activation opened K_*Ca*_1.1 single-channel current (Gebremedhin et al., 2017). In mouse mesenteric arteries, approximately 50% of the EET-induced vasodilation is reported to be endothelium-independent due to its direct action on the smooth muscle TRPV4-K_*Ca*_1.1 axis (Earley et al., 2009). In rat retinal arterioles, GSK1016790A activates vasodilation in NO- and K_*Ca*_1.1 channel-dependent manners *in vivo* (Mori et al., 2020). In the cerebral basal artery of cerebral ischemia-reperfusion injury rats, the vasorelaxation induced by the total flavonoids of rhododendron is linked to the TRPV4-K_*Ca*_1.1 pathway in smooth muscle cells (Han et al., 2018). Since TRPC1 associates with the K_*Ca*_1.1 channel to form a signal complex in vascular smooth muscles, controlling smooth muscle hyperpolarization and vascular tone (Kwan et al., 2009), the TRPV4-TRPC1-KCa1.1 complex is probably present in vascular smooth muscle. It has been reported that in isolated human left internal mammary arteries segments, 11,12-EET induces smooth muscle membrane hyperpolarization and vascular relaxation through the TRPV4-TRPC1-KCa1.1 complex (Ma et al., 2015), suggesting the involvement of the TRPV4-TRPC1-KCa1.1 complex in smooth muscle-mediated vascular dilation.

**FIGURE 2:**
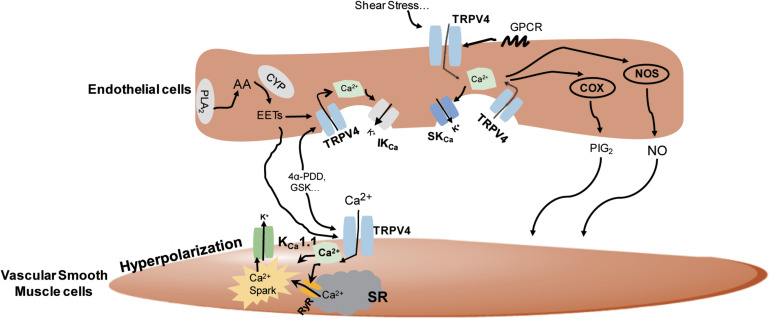
The underlying mechanisms of the regulation of TRPV4 in vasodilation. In the vascular endothelial cells, TRPV4 can be activated by stimuli-sensing, G protein-coupled receptors (GPCR) activation, epoxyeicosatrienoic acid (EETs), GSK1016790A (GSK), and 4α-PDD. Its activation leads to Ca^2+^ influx and the subsequent activation of nitric oxide synthase (NOS), cyclooxygenase (COX), and intermediate-conductance Ca^2+^-sensitive K^+^ channels/small conductance Ca^2+^-sensitive K^+^ channels (IK_*Ca*_/SK_*Ca*_) channels to induce nitric oxide (NO), prostacyclin (PGI_2_), and endothelial-derived hyperpolarizing factor (EDHF) production, respectively, and causes vasodilation. In smooth muscles, TRPV4 can be activated by endothelial-derived EETs or chemical agonists, leading to Ca^2+^ entry-induced Ca^2+^ release from ryanodine receptor (RyR) in the sarcoplasmic reticulum (SR), the stimulation of Ca^2+^ sparks, and the subsequent activation of K_*Ca*_1.1 to stimulate smooth muscle hyperpolarization and vasodilation.

In summary, the TRPV4 channels can be activated by a wide range of stimuli and regulate vasorelaxation in both endothelium-dependent and endothelium-independent manners involving complicated distinct pathways (Figure 2).

## Transient Receptor Potential Vanilloid 4 and Vasoconstriction

In addition to the well-documented vasorelaxation, TRPV4 channel activation can evoke constriction in some vascular beds (Saifeddine et al., 2015; Dryn et al., 2016; Zhang et al., 2018). Physiologically, TRPV4 channels participate in the regulation of the adrenergic vascular contractile activity of pulmonary artery smooth muscle cells (PASMCs) (Dryn et al., 2016). TRPV4 is shown to participate in the PASMC contractile response to hypoxia as the inhibition of TRPV4 blocks hypoxia-induced Ca^2+^ influx and myosin light chain phosphorylation in human PASMCs (Goldenberg et al., 2015b). 4α-PDD induces extracellular Ca^2+^ entry and TRPV4 overexpression increases basal [Ca^2+^]_*i*_ in hypoxic PASMCs, resulting in myogenic tone enhancement and the development of hypoxia-induced pulmonary hypertension (Yang et al., 2012). Moreover, TRPV4 contributes to 5-hydroxytryptamine (5-HT)-mediated pulmonary vasoconstriction in chronic hypoxic pulmonary hypertension (Xia et al., 2013). A recent study find that the inhibition of TRPV4 reverses steady-state myogenic tone and inhibits the pressure-evoked membrane depolarization, [Ca^2+^]_*i*_ increase and constriction in neonatal pig pre-glomerular distal interlobular arteries (Soni et al., 2017). In addition, the blockade of TRPV4 channels attenuates angiotensin II (Ang II)-induced Ca^2+^ entry and constriction in the pre-glomerular microvessels of neonatal pigs (Soni et al., 2019).

Pathologically, the action of TRPV4 contributes to vascular contraction under some specific conditions. For example, in mouse aorta, the G protein-coupled receptor (GPCR) potentiation of TRPV4 action leads to vasoconstriction through COX-dependent prostanoid production and Tx receptor activation (Saifeddine et al., 2015). Additionally, in mouse aortas from hypertensive mice, GSK1016790A-activated TRPV4 channels increase [Ca^2+^]_*i*_, resulting in endothelium-dependent contraction through the activation of cytosolic phospholipase A2 (cPLA2) and COX2 (Zhang et al., 2018). These studies suggest that TRPV4 channel activation can increase [Ca^2+^]_*i*_ and generate vasoconstricting prostanoids to constrict vessels under some specific pathological conditions.

## Transient Receptor Potential Vanilloid 4 and Vascular Permeability

The current knowledge suggests that TRPV4 participates in the control of vascular permeability, particularly in the lung capillary endothelial barrier. The activation of TRPV4 channels is shown to increase lung epithelial-endothelial permeability and disrupt alveolar walls (Alvarez et al., 2006). TRPV4 activation causes Ca^2+^-dependent vascular hyperpermeability in lung, leading to the disruption of the alveolar septal barrier and acute lung injury following high-pressure ventilation (Hamanaka et al., 2007). It has been demonstrated that in human and mouse lung microvascular endothelial cells, exogenous H_2_O_2_ increases [Ca^2+^]_*i*_ and barrier permeability via activation of TRPV4 through the Src kinase Fyn (Suresh et al., 2015). TRPV4 agonist 4α-PDD causes endothelial blebs and/or breaks in rat and mouse lungs, and increases lung endothelial permeability in a Ca^2+^-entry dependent manner (Alvarez et al., 2006). Mechanically, 4α-PDD induces pulmonary arterial endothelial cell barrier disruption through the mitochondrial redistribution of uncoupled eNOS secondary to a protein kinase C-dependent phosphorylation of eNOS at Threonine 495 (T495) (Lu et al., 2021). TRPV4 inhibition or deletion ameliorates endothelial permeability and fluid-induced lung injury, involving the release of Ang II and P-selectin (Bihari et al., 2017). The TRPV4 channels are also involved in the Pannexin 1-regulated lung vein permeability (Maier-Begandt and Comstra, 2021) and the vascular hyperpermeability induced by GPCR protease-activated receptor 1 (PAR1) in the airways and upper gastrointestinal tract of mice (Peng et al., 2020). In addition, deletion of the *TRPV4* gene reduces colonic vascular endothelial permeability during dextran sulfate sodium-induced murine colonic inflammation, whereas the up-regulation of TRPV4 results in the progression of colonic inflammation by increasing vascular permeability (Matsumoto et al., 2018). These data indicates the importance of TRPV4 for vascular permeability, providing targetable pathways to regulate vascular permeability and barrier function, and to prevent inflammation-induced harmful effects of vascular leak.

## Transient Receptor Potential Vanilloid 4 and Vascular Remodeling

TRPV4 participates in vascular remodeling regulation mainly by controlling vasculogenesis and arteriogenesis (Negri et al., 2019). TRPV4 expression is significantly increased in fluid shear stress-stimulated cerebral collateral circulation and 4α-PDD-induced TRPV4 activation enhances cerebral arteriogenesis (Schierling et al., 2011), while chronic hypoxia-induced vascular remodeling and right ventricle hypertrophy are blocked in a TRPV4-deficient mouse model (Yang et al., 2012), confirming the vital role of TRPV4 in vascular remodeling regulation. Numerous Ca^2+^-dependent transcription factors, such as nuclear factor of activated T cells, myocyte enhancer factor 2C, and calcineurin-dependent 1 are involved in the regulation of TRPV4 in arteriogenesis (Troidl et al., 2010). TRPV4 plays a key role in cytoskeletal reorganization and cell adhesion, which regulates endothelial cell proliferation and motility through mechanotransduction (Reddy et al., 2015; Adapala et al., 2016; Thoppil et al., 2016). The cyclical stretching-evoked stimulation of TRPV4 mediates cytoskeletal remodeling and cell reorientation through integrin-to-integrin signaling (Thodeti et al., 2009). TRPV4 participates in the calcium oscillations and VSMC migration induced by platelet-derived microvesicles (PMVs) (Li et al., 2021), implying that TRPV4 promotes VSMC migration under pathological conditions resulting in vascular remodeling regulation. Besides VSMCs, TRPV4 has also been known to regulate neovascularization by inducing endothelial cell proliferation and migration (Moccia, 2018). It is found that 4α-PDD-activated TRPV4 induces Ca^2+^ entry and proliferation in human brain capillary endothelial cells (Fiorio Pla et al., 2012) and such proliferation is partially inhibited by TRPV4-siRNA (Hatano et al., 2013), suggesting that endothelial TRPV4 partially mediates cell proliferation. A recent study in isolated lung microvascular endothelial cells from rats subjected to SU5416 [an inhibitor of vascular endothelial growth factor receptor-2 (VEGFR2)] plus hypoxia shows that endogenous ROS increases the basal levels of [Ca^2+^]_*i*_ via TRPV4 activation and promotes aberrant migratory and proliferative capacity (Suresh et al., 2018). Evidence also indicates that 4α-PDD causes endothelial cell proliferation, triggering collateral vessel growth (Troidl et al., 2009). In addition, 4α-PDD decreases the infarct volume, improves the recovery of neurological function, and enhances vasculogenesis and neurogenesis in ischemic stroke rats (Chen et al., 2018). These results suggest that both VSMCs and endothelial cells play important roles in TRPV4-regulated vascular remodeling.

It is reported that TRPV4 negatively regulates tumor angiogenesis and tumor vessel maturation (Adapala et al., 2016). For instance, in tumor endothelial cells, the deletion of the *TRPV4 g*ene induces cell proliferation, migration, sprouting angiogenesis, and abnormal tube formation *in vitro* accompanied by an increase in basal Rho activity (Thoppil et al., 2016). In contrast, the activation or overexpression of TRPV4 restores aberrant migration and angiogenesis by inhibiting the exacerbated Rho activity (Adapala et al., 2016). Additionally, a study on TRPV4 knockout tumors shows that silencing of the *TRPV4* gene stimulates VEGF-regulated migration *in vitro* and increases the expression of VEGFR2 *in vivo* in the vasculature compared to control tumors (Kanugula et al., 2019). A recent study with endothelial-specific TRPV4 knockout mice demonstrates that the specific deletion of endothelial TRPV4 promotes tumor angiogenesis, growth, and metastasis compared to control mice (Kanugula et al., 2021), indicating that endothelial TRPV4 is a critical modulator of vascular integrity and tumor angiogenesis. The discrepant function of TRPV4 in vascular remodeling probably is dependent on the cell type and animal model used in different studies.

## Transient Receptor Potential Vanilloid 4 and Vascular Damage

Reactive oxygen species production has been implicated in the pathogenesis of atherosclerosis, ischemia/reperfusion injury, obstructive sleep apnea, and other diseases, playing a critical role in vascular physiology and pathophysiology (Droge, 2002). GSK1016790A increases phosphorylation of eNOS and adenosine 3′,5′-monophosphate-activated protein kinase in the aorta and decreases leukocyte adhesion to tumor necrosis factor α-inflamed endothelium, and oral administration of GSK1016790A reduces atherosclerotic plaque formation in ApoE deficient mice with a western-type diet, suggesting that pharmacological activation of TRPV4 may serve as a potential therapeutic approach to treat atherosclerosis (Xu et al., 2016). However, recent study shows that genetic deletion or chemical antagonism of TRPV4 channels blocks lipopolysaccharide-triggered exacerbation of oxidized low-density lipoprotein (oxLDL)-mediated foam cell formation (Gupta et al., 2019), and TRPV4 deficiency prevents pathophysiological range matrix stiffness or scratch-induced exacerbation of oxLDL-induced foam cell formation (Goswami et al., 2017). Therefore, it still needs to deeply clarify the role of TRPV4 in atherogenesis development.

In addition, TRPV4 is a critical mediator of pressure-induced damage related to ventilator-induced lung injury, infarction, and heart failure (Balakrishna et al., 2014). GSK1016790A decreases right ventricular and systemic pressure, resulting in fatal circulatory collapse, accompanied by increased protein permeability, lung hemorrhage and fluid extravasation (Wandall-Frostholm et al., 2015). TRPV4 inhibition prevents edema and inflammation, and improves oxygen saturation and pulmonary function in HCl- and Cl_2_-induced chemical lung injury, suggesting TRPV4 inhibitors as a potential treatment for acute lung injury (Balakrishna et al., 2014). Furthermore, the inhibition of TRPV4 channels increases the viability of astrocytes against oxidative stress induced by mercaptosuccinate or buthionine sulfoximine (Bai and Lipski, 2010). It is suggested that after traumatic brain injury, excessive mitochondria-derived H_2_O_2_ activates BK_*Ca*_ channels via a TRPV4-dependent pathway in the VSMCs, impairing pressure-induced constriction of cerebral arteries (Szarka et al., 2018). The up-regulation or activation of TRPV4 contributes to endothelial damage leading to tissue damage, vascular destabilization, and blood-spinal cord barrier breakdown after spinal cord injury, whereas the knockdown or inhibition of TRPV4 prevents such effects (Kumar et al., 2020), indicating that the regulation of TRPV4 signaling might lead to new therapeutic strategies to protect endothelial cells and enhance repair after spinal cord injury.

## Transient Receptor Potential Vanilloid 4 as a Potential Therapeutic Target

TRPV4 channels are widely expressed in various tissues, playing important roles in many physiological and pathophysiological processes (Goldenberg et al., 2015a; Randhawa and Jaggi, 2015a). The activation of TRPV4 channels has been implicated in pulmonary hypertension (Suresh et al., 2018), hyponatremia (Carreno et al., 2009; Tian et al., 2009), neurodegenerative skeletal muscle dysplasia (Auer-Grumbach et al., 2010), and bone disorders (Mizoguchi et al., 2008) and its inhibition presents a potential therapeutic strategy for pain, gastrointestinal disorders, edema, and lung diseases (Grace et al., 2017). Therefore, TRPV4 channels become a potential therapeutic target in numerous diseases.

Increasing evidence has indicated that TRPV4 participates in the regulation of systemic vasculature reactivity and the maintenance of cardiovascular homeostasis (Randhawa and Jaggi, 2015b). TRPV4 plays an important role in blood pressure control. The activation of TRPV4 stimulates the relaxation of peripheral resistance arteries and regulates arterial pressure (Earley et al., 2009). TRPV4 activity is markedly impaired in Ang II-induced hypertensive mice (Sonkusare et al., 2014). TRPV4 channels probably function as a compensatory pathway to induce vasorelaxation under the condition of NO blockage (Feletou and Vanhoutte, 2006). Thus, TRPV4 might become a therapeutic target in hypertension treatment. Additionally, TRPV4 is involved in converting mechanical forces on the airways to molecular and transcriptional events, regulating the development of lung and stabilization of pulmonary vasculature (Morgan et al., 2018). Recent evidence suggests that activating pulmonary capillary endothelial TRPV4 channels enhance pulmonary venous pressure-induced edema and TRPV4 blockade prevents the increased vascular permeability and pulmonary edema (Thorneloe et al., 2012), highlighting a pharmacological therapeutic potential of TRPV4 inhibition for pulmonary edema induced by heart failure. The TRPV4 antagonist GSK2193874 mitigates the increases in lung weight after heart failure (Michel, 2006; Thorneloe et al., 2012) and TRPV4 inhibition or genetic deletion suppresses pulmonary inflammation and improved pulmonary function in the HCl-and Cl_2_- induced injury model (Balakrishna et al., 2014). These data indicate that TRPV4 might be a therapeutic target for pulmonary disease. It is showed that matrix stiffness promotes foam cell formation in a TRPV4-dependent manner by regulating the uptake of oxLDL in macrophages, suggesting the importance of TRPV4 channels for development of atherosclerotic lesions (Goswami et al., 2017). Therefore, targeting TRPV4 might be a potential therapeutic tactic to treat atherosclerosis.

Apart from cardiovascular diseases, TRPV4 has been found to affect diabetes-related complications. Recent studies have shown that TRPV4 is implicated in the replication of β-cells, and the production and secretion of insulin (Skrzypski et al., 2013). TRPV4 agonists exert insulinotropic effects on pancreatic β-cell lines, but extend activation resulted in cell dysfunction and death (Billert et al., 2017). TRPV4 agonists promote vasorelaxation and improve cardiovascular function in a rodent type 2 diabetes model, and TRPV4 antagonists reduce high-fat diet-induced obesity, insulin resistance, and diabetes-related complications (Hu et al., 2020), suggesting its potential as a therapeutic target for such diseases. Gastrointestinal inflammation increases TRPV4 expression and enhances channel activation (D’Aldebert et al., 2011; Cenac et al., 2015), and TRPV4 inhibitors demonstrate therapeutical potential for treating inflammation and abdominal pain (Holzer, 2011). Research has suggested that blocking TRPV4 activity might be a strategy for scleroderma treatment (Sharma et al., 2017). TRPV4 channels participate in the KCa3.1-regulated proliferation of human bronchial smooth muscle cells in the process of chronic asthma (Yu et al., 2017), indicating that it is a potential therapeutic target for chronic asthma treatment. TRPV4 has also been determined to have a pathological role in the activation and proliferation of hepatic stellate cells (Song et al., 2014; Zhan and Li, 2018), suggesting a potential therapeutic target for liver fibrosis treatment. Following cerebral hypoxia and ischemia, TRPV4 expression and activity are enhanced in hippocampal astrocytes, indicating the involvement of TRPV4 in the pathogenesis of astroglial reactivity after an ischemic insult (Butenko et al., 2012).

Some safety concerns about global TRPV4 agonism or antagonism have been raised as TRPV4 have both favorable and unfavorable effects on the same tissue. For example, TRPV4 is essential for endothelial barrier integrity, and TRPV4 inhibitors could be beneficial for pulmonary edema. However, the primary role of TRPV4 in hypoxic pulmonary vasoconstriction indicates the detriment of TRPV4 inhibition to patients with lung disease (Yang et al., 2012; Morty and Kuebler, 2014; Goldenberg et al., 2015a). Additionally, TRPV4 inhibition also represents a much-needed “two-pronged attack” against ARDS, for its barrier-stabilizing effect coupled with anti-inflammatory properties (Goldenberg et al., 2015a). The therapeutic target potential of TRPV4 is currently being studied in the clinical trial with a first-in-class, highly potent, selective TRPV4 channel blocker GSK2798745 from GlaxoSmithKline (Moran, 2018) (Goyal et al., 2019). A recent clinical trial study shows that GSK2798745 does not affect lipopolysaccharide-induced elevation of barrier permeability and inflammation (Mole et al., 2020). Because discrepant effect of TRPV4 on many tissues and systemic TRPV4 blockage may be detrimental, targeting the signaling pathways upstream of TRPV4 activation might be an alternative strategy.

## Summary

The dysfunction of TRP channels is associated with endothelial dysfunction, which is reflected by NO bioavailability decrease, vascular smooth muscle tonicity dysregulation, endothelial barrier dysfunction, oxidative damage increase, and angiogenic disorder. The current review summarizes reports about the role of TRPV4 in vascular dilation and constriction, vascular permeability, vascular remodeling, and vascular damage, and discusses possible mechanisms. TRPV4 channels can be activated by a wide range of stimuli by means of distinct pathways and its impact on regulating vascular function is shown to vary between vessels and pathological conditions. The activation of TRPV4 channels contributes to vasodilation involving endothelial NO, PGI_2_, and EDHF pathways, or directly cause VSMC hyperpolarization and vasodilation. On the contrary, in some specific vascular beds or under specific pathological conditions, TRPV4 activation evokes constriction. TRPV4 also participates in the control of the lung capillary endothelial barrier, lung injury and vascular remodeling regulation. Since the complicated and discrepant function of TRPV4 in same tissues, more studies about cell-type specific role of TRPV4 are required to fully understand its pathobiological and beneficial potential under various pathophysiological conditions in the future. Additionally, the upstream of TRPV4 regulation is still need to be more investigated, as well as the precise function switching and underlying mechanism on the same tissues. Given that TRPV4 has equivocal effects on the same tissue, the safety concerns about global TRPV4 agonism or antagonism need to be studied. Therefore, whether agents targeting TRPV4 clinically have dual impacts on the regulation of vascular function in humans under different pathological conditions remains to be determined.

## Author Contributions

LL, MG, XL, ZW, and TZ performed document searching and reviewing. LL, MG, JY, YL, and FY contributed to literature review. XW and LF provided a critical reading of the manuscript. TZ initiated the project and wrote the manuscript. All authors read and approved the final version of the manuscript.

## Conflict of Interest

The authors declare that the research was conducted in the absence of any commercial or financial relationships that could be construed as a potential conflict of interest.

## Abbreviations

ACh: acetylcholine
Ang: angiotensin
ARDS: acute respiratory distress syndrome
[Ca^2+^]_*i*_: intracellular Ca^2+^ concentration
COX: cyclooxygenase
cPLA2: cytosolic phospholipase A2
EDHF: endothelial-derived hyperpolarizing factor
EET: epoxyeicosatrienoic acid
GPCR: G protein-coupled receptors
IK_*Ca*_: intermediate-conductance Ca^2+^-sensitive K^+^ channels
K_*Ca*_1.1: large-conductance Ca^2+^-activated K^+^ channel
NO: nitric oxide
NOS: nitric oxide synthase
PGI_2_: prostacyclin
PAR1: protease activated receptor 1
PASM: pulmonary artery smooth muscle
ROS: reactive oxygen species
RyR: ryanodine receptor
SR: sarcoplasmic reticulum
SK_*Ca*_: small conductance Ca^2+^-sensitive K^+^ channels
TRP: transient receptor potential
TRPV4: transient receptor potential vanilloid 4
VEGFR: vascular endothelial growth factor receptor
VSMC: vascular smooth muscle cell
4 α-PDD: 4 α-phorbol 12,13-didecanoate
5-HT: 5-hydroxytryptamine.
